# Fast Blood Oxygenation through Hemocompatible Asymmetric Polymer of Intrinsic Microporosity Membranes

**DOI:** 10.34133/research.0151

**Published:** 2023-05-19

**Authors:** Xinxi Huang, Junping Huang, Pengcheng Su, Wanbin Li

**Affiliations:** Guangdong Key Laboratory of Environmental Pollution and Health, School of Environment, Jinan University, Guangzhou 511443, China.

## Abstract

Membrane technology has attracted considerable attention for chemical and medical applications, among others. Artificial organs play important roles in medical science. A membrane oxygenator, also known as artificial lung, can replenish O_2_ and remove CO_2_ of blood to maintain the metabolism of patients with cardiopulmonary failure. However, the membrane, a key component, is subjected to inferior gas transport property, leakage propensity, and insufficient hemocompatibility. In this study, we report efficient blood oxygenation by using an asymmetric nanoporous membrane that is fabricated using the classic nonsolvent-induced phase separation method for polymer of intrinsic microporosity-1. The intrinsic superhydrophobic nanopores and asymmetric configuration endow the membrane with water impermeability and gas ultrapermeability, up to 3,500 and 1,100 gas permeation units for CO_2_ and O_2_, respectively. Moreover, the rational hydrophobic–hydrophilic nature, electronegativity, and smoothness of the surface enable the substantially restricted protein adsorption, platelet adhesion and activation, hemolysis, and thrombosis for the membrane. Importantly, during blood oxygenation, the asymmetric nanoporous membrane shows no thrombus formation and plasma leakage and exhibits fast O_2_ and CO_2_ transport processes with exchange rates of 20 to 60 and 100 to 350 ml m^−2^ min^−1^, respectively, which are 2 to 6 times higher than those of conventional membranes. The concepts reported here offer an alternative route to fabricate high-performance membranes and expand the possibilities of nanoporous materials for membrane-based artificial organs.

## Introduction

Artificial organs can partially or completely perform the functions of human organs that are temporarily or permanently disabled, and play important roles in medical science. A membrane oxygenator, also known as artificial lung, can remove CO_2_ and replenish O_2_ for blood to maintain vital signs during surgery [1]. An extracorporeal membrane oxygenator (ECMO) provides imperative clinical support for patients with cardiopulmonary impairment, respiratory failure, cardiogenic shock, etc. For example, it has been proven to have a positive effect on the survival rate of COVID-19-infected patients with acute respiratory failure [2]. As a key component, the lung membrane directly comes into contact with blood to realize gas exchange and determine effectiveness. A membrane has to possess the following characteristics: (a) sufficient gas permeation capacity to allow fast exchange, thereby reducing effective membrane area and prefilling blood volume; (b) impermeability for plasma to avoid leakage, which seriously deteriorates oxygenation efficiency and threatens patient survival; and (c) excellent hemocompatibility to restrain thrombosis and hemolysis. Other features, e.g., processability, should also be taken into account.

Many types of materials, including polypropylene (PP), polysulfone (PSF), polydimethylsiloxane (PDMS), and polymethylpentene (PMP), have been employed to fabricate membranes for oxygenation [3]. Among these, PP membranes are efficient for exchange, yet the existence of pores of tens of nanometers brings about a wetting issue despite their hydrophobicity, and then leads to plasma leakage and efficiency attenuation, especially in operations lasting over several hours [4]. Siloxanes, e.g., PDMS, have been coated on membranes to prevent leakage [5]. PDMS membranes allow gas transport with a permeability of hundreds of Barrer (1 Barrer = 3.35 × 10^−16^ m mol m^−2^ s^−1^ Pa^−1^), but their thickness (several micrometers) limits gas permeance. PMP membranes have an intrinsic O_2_ permeability of only 30 Barrer, but their asymmetric structure with skin layers enables satisfactory permeation and antiwetting properties [6]. Nonetheless, their permeance is probably insufficient and the CO_2_/O_2_ permselectivity is distinct from that of alveoli [7]. For hemocompatibility, when blood comes into contact with membranes, proteins may be adsorbed, thus promoting platelet adhesion and activation, resulting in coagulation, immune response, and thrombus formation [8,9]. To improve hemocompatibility, biomedical and biomimetic substances, e.g., heparin and polyphenol, have been immobilized to form antithrombotic surfaces [3,10]. Grafting surfaces by hydrophobic or hydrophilic reagents, which is still uncertain, is proposed to eliminate or suppress protein and platelet attachments as well [10–14]. Some emerging polymers show good hemocompatibility, but no data about oxygenation have been presented [11,13,15]. Hemolysis, mainly affected by ECMO circuits [16] and probably influenced by membrane morphology, surface chemistry, etc., is another hemocompatible issue. It is extremely challenging to obtain an oxygenation membrane with great exchange performance and hemocompatibility.

**Fig. 1.**
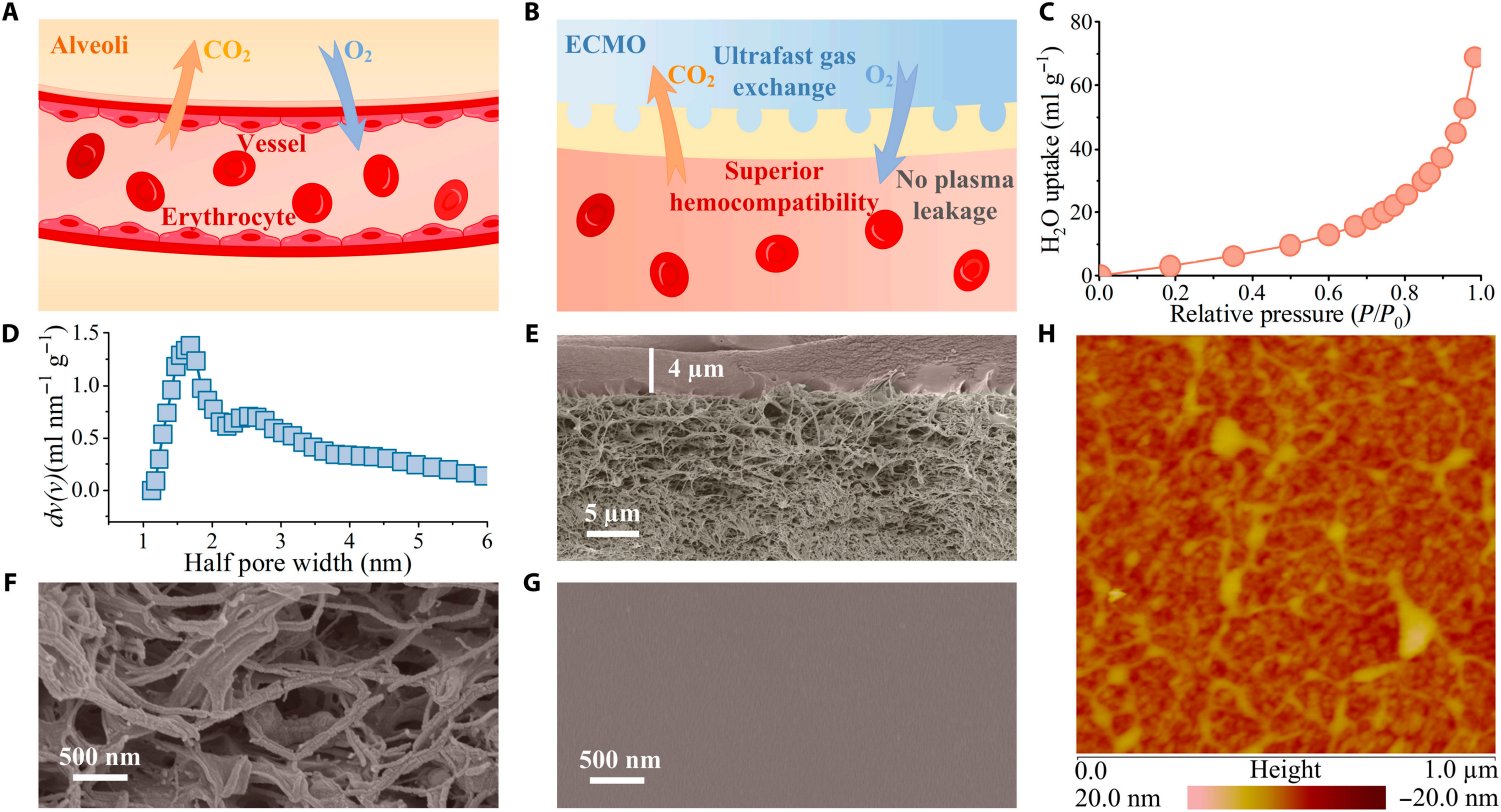
Asymmetric nanoporous membranes for oxygenation. (A) Schematic of gas exchange at alveoli. (B) Schematic of blood oxygenation in an AsNp membrane lung. Color change of erythrocyte from dark red to bright red represents blood oxygenation. (C) Water vapor isotherm of PIM-1. (D) Pore width distribution of PIM-1. (E) Cross-sectional SEM image of the AsNp membrane with a standing time of 1 min. (F) Cross-sectional SEM image of porous sublayer of the AsNp membrane. (G) Top view SEM image of the AsNp membrane. (H) AFM image of the AsNp membrane.

Herein, we report an asymmetric nanoporous (AsNp) membrane, made of polymer of intrinsic microporosity-1 (PIM-1) via the classic nonsolvent-induced phase separation (NIPS) method, for blood oxygenation (Fig. 1A and B). Different from conventional porous and nonporous membranes suffering from plasma leakage and inferior permeability, the AsNp membrane with superhydrophobic nanopores and asymmetric structure is water impermeable and gas ultrapermeable. Moreover, the appropriate hydrophobic–hydrophilic nature, charged feature, and roughness of surface endow the membrane with inhibited protein adsorption, coagulation, and hemolysis. In addition, for blood oxygenation, the AsNp membrane shows competitive performance, with rapid O_2_ and CO_2_ exchange and without detectable thrombus.

## Results

### Asymmetric nanoporous membranes

We synthesized the yellow PIM-1 powder by spirobisindane-tetrafluoroterephthalonitrile polycondensation [17]. As a type of microporous polymer, the ladder-like and rigid chains give the polymer numerous interconnected nanopores [17–19]. Nitrogen adsorption–desorption isotherms and pore width distribution indicated the high porosity of PIM-1 with a specific surface area of 790 m^2^ g^−1^ calculated by the Brunauer–Emmett–Teller (BET) theory and a uniform nanopore diameter of 1.7 nm from the slit-pore model-based nonlocal density functional theory (Fig. 1C and D and Fig. S1). Water vapor isotherm (Fig. 1C), with a low uptake even close to saturation pressure, was a typical curve for the porous materials containing superhydrophobic nanopores [20]. Such pore properties were conducive to gas permeability and plasma impermeability of membranes. PIM-1 membranes are typically prepared by solvent evaporation phase conversion and have a thickness of tens to hundreds of micrometers, which restricts their permeance [18,21]. Asymmetric membranes have thin skin layers [22,23]. Here, NIPS with a chloroform solvent and a methanol nonsolvent was developed to construct the AsNp membranes. Through casting the PIM-1 chloroform ink on a polytetrafluoroethylene substrate (PTFE) (Figs. S2 to S6), the AsNp composite membrane was formed by simply standing at atmosphere and quenching in methanol. As illuminated in scanning electron microscopy (SEM) images (Fig. 1E to G), the AsNp membrane consisted of a skin layer and a porous sublayer. Solvent chloroform was evaporated during atmosphere standing to form dense shin layer. When the casted layer was immersed in a coagulant bath, the solvent exchange between nonsolvent and solvent, i.e., methanol and chloroform, migrated into and out of the PIM-1 matrix, respectively, and induced phase separation and solidification. With the shortening of evaporation time, the skin layers became thinner (Figs. S2 to S5); e.g., the AsNp membrane with a standing time of 1 min had a thickness of ~4.0 μm (Fig. 1E), which was an order of magnitude smaller than that of conventional PIM-1 membranes with an entirely dense structure [17,20]. An atomic force microscopy (AFM) image illustrated a smooth surface with a root mean square roughness of 2.1 nm and an arithmetic average roughness of 1.6 nm, from a probe area of 1 × 1 μm^2^ (Fig. 1H). A smoother surface usually meant less protein adsorption and mechanical friction to blood cells.

### Gas ultrapermeability and water impermeability

**Fig. 2.**
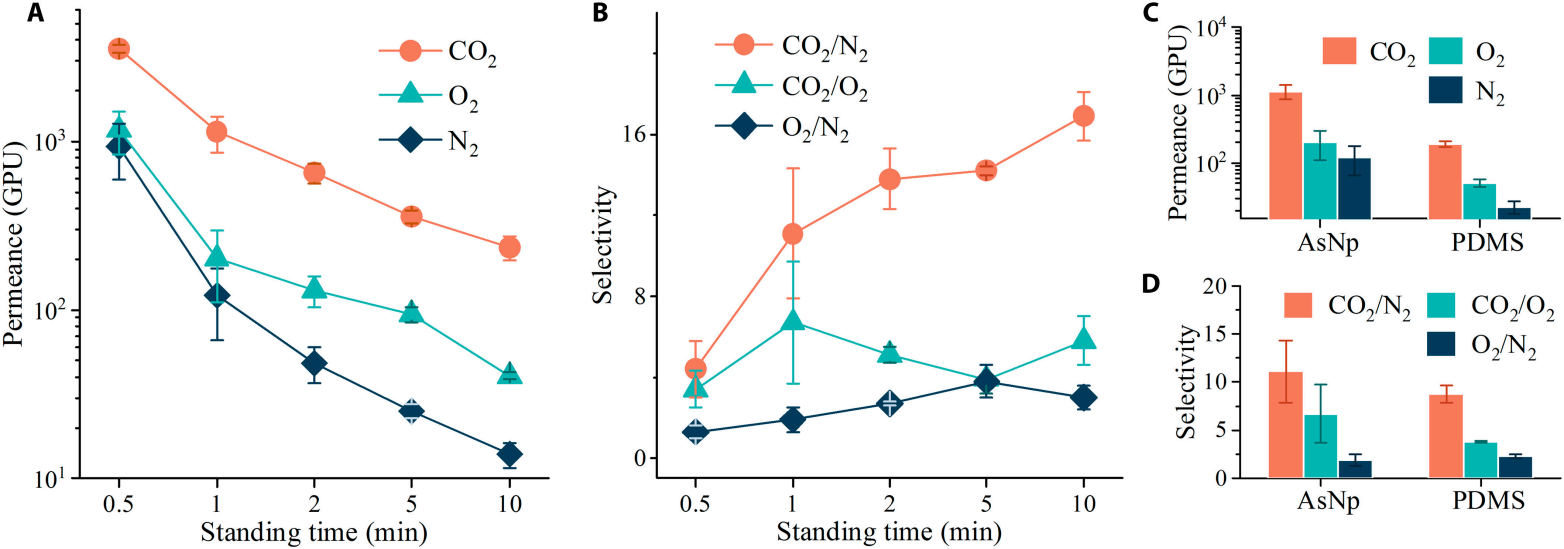
Permeation property of AsNp membranes. (A) CO_2_, O_2_, and N_2_ permeances of the AsNp membranes with different standing times. (B) CO_2_/N_2_, CO_2_/O_2_, and O_2_/N_2_ selectivities of the AsNp membranes with different standing times. (C and D) Comparison between the AsNp membrane with a standing time of 1 min and the PDMS membrane for gas permeance and selectivity, respectively.

Transport behaviors of CO_2_, O_2_, and N_2_ through the AsNp membranes are measured and presented in Fig. 2A and B. Gas permeation in polymer membranes is related to molecular sorption/solution and diffusion [24]. Sorption is determined by gas condensability and its affinity to membrane, and diffusion is governed by gas kinetic diameter and membrane pore size. Benefitting from the preferential adsorption of PIM-1 toward CO_2_ [25], the CO_2_/O_2_ and CO_2_/N_2_ selectivities of the AsNp membrane with a standing time of 1 min were 6.7 and 11.1, respectively, which were larger than those of PMP membranes [7,11]. Relative to the entirely dense PIM membrane with high CO_2_ permeability but moderate permeance [21], the AsNp membrane exhibited a CO_2_ permeance of 1,137 gas permeation units (1 GPU = 3.35 × 10^−10^ mol m^2^ s^−1^ Pa^−1^) due to the thin skin layer. Meanwhile, the O_2_ permeance was achieved at 203 GPU. Considering the thickness of the selective layer, the permeability of AsNp was poorer than the intrinsic one, implying the transfer resistance from the sublayer. As the thickness decreased, the permeance and CO_2_/N_2_ selectivity were enhanced and deteriorated, respectively. For example, the AsNp membrane with a standing time of 0.5 min had CO_2_ and O_2_ permeances of 3,545 and 1,169 GPU, respectively. We prepared the classic PDMS and PSF membranes and measured their permeation property for comparison (Fig. 2C and D and Figs. S7 to S9). The permeance and selectivity of the PDMS membrane were good but still obviously inferior to those of AsNp. We studied the aging behavior of the AsNp membrane (Fig. S10). After aging for 270 days, the CO_2_ permeance (600 GPU) of AsNp was higher than that of PDMS as well. Apart from gas permeation, water inaccessibility is critical, especially for oxygenation with pressure drop at the blood side. Unlike the porous PSF membrane having SEM-detectable pores and being permeable to water (Figs. S8 and S11), the AsNp membrane with superhydrophobic nanopores was inaccessible to water like the PDMS membrane, even at a transmembrane pressure of 0.4 MPa and after filtration for several hours (Fig. S11). The above features revealed that the AsNp membrane was promising for eliminating plasma leakage and performing fast gas exchange.

### Superior hemocompatibility

**Fig. 3.**
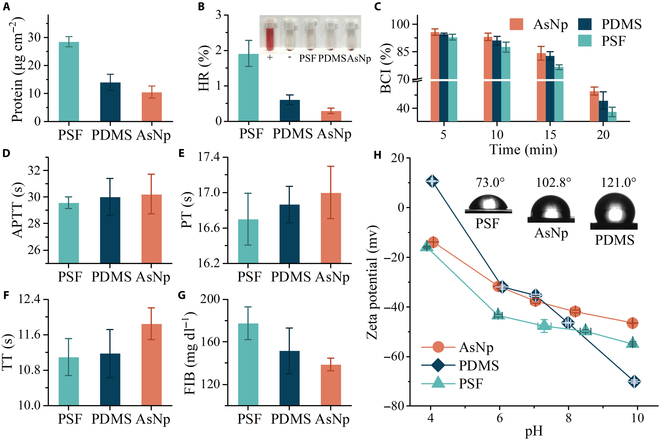
Hemocompatibility of AsNp membranes. (A) Protein adsorption of the PSF, PDMS, and AsNp membranes. (B) Hemolysis ratio (HR) of the PSF, PDMS, and AsNp membranes. Inset photographs show the positive control, negative control, and erythrocyte solutions after incubation of PSF, PDMS, and AsNp for 1 h. (C) Blood clotting index (BCI) of the PSF, PDMS, and AsNp membranes with time extending. (D) Activated partial thromboplastin time (APTT) of the PSF, PDMS, and AsNp membranes. (E) Prothrombin time (PT) of the PSF, PDMS, and AsNp membranes. (F) Thrombin time (TT) of the PSF, PDMS, and AsNp membranes. (G) Fibrinogen (FIB) content of the PSF, PDMS, and AsNp membranes. (H) Water contact angle and Zeta potential of the PSF, PDMS, and AsNp membranes.

Some typical experiments, protein adsorption, platelet adhesion and activation, hemolysis, and clotting time, were performed to investigate hemocompatibility. Protein adsorption on foreign materials initiates coagulation cascade and thrombosis. Bovine serum albumin, a representative protein, adsorbed on AsNp with an amount of 10.5 μg cm^−2^, which was lower than that of the widely biologically used PSF and PDMS and other biomedical materials (Fig. 3A and Fig. S12) [3,11,13]. Platelet adhesion, activation, and clotting time were tested by using sheep blood. In contrast to PSF and PDMS showing few fluorescent speckles from platelet aggregation, almost no speckle could be observed on the AsNp membrane surface (Fig. S12D to F). The irregular platelets on PSF and PDMS indicated platelet activation, whereas the platelet adhered to AsNp kept the morphology without pseudopodia or deformation (Fig. S12D to F, insets). Hemolysis usually refers to erythrocyte rupture and hemoglobin (HGB) release and will cause a reduction in exchange efficiency and will have a toxic effect. After incubation in erythrocyte solution for 1 h, the hemolysis ratio (HR) of AsNp analyzed by HGB was only 0.29% (Fig. 3B). This value could meet the clinical requirement (2.0%) according to American Society for Testing and Materials standard and was much less than that of the PSF and PDMS membranes. Blood clotting index (BCI) denotes that blood components still disperse in plasma rather than adhere to membranes after clotting. Clearly, the AsNp membrane had a higher BCI than PDMS and PSF as time prolonged (Fig. 3C). Activated partial thromboplastin time (APTT), prothrombin time (PT), and thrombin time (TT) reflect intrinsic, extrinsic, and common coagulation pathways, respectively. The APTT, PT, and TT of AsNp (30.22 s, 17.00 s, and 11.85 s) were greater than those of PDMS and PSF (Fig. 3D to F). The fibrinogen (FIB) content (138.8 mg dl^−1^) of AsNp was lower than that of PDMS and PSF (Fig. 3G), which could reduce fibrin formation and inhibit the occurrence of blood coagulation. All the above experimental results suggested the great anticoagulation and antithrombotic properties of the AsNp membrane.

Hemocompatibility correlates with various surface physicochemical properties of membranes. Both hydrophilic and hydrophobic modifications are applied to improve hemocompatibility, which drives the formation of a hydration layer or a low-energy interface and then relieves protein and cell attachments [10–14]. Compared with the other 2 membranes, the AsNp membrane showed moderate water (102.8^o^) and blood (101.1^o^) contact angles (Fig. 3H and Fig. S13A and B). In view of the superhydrophobicity of inner nanopores, the AsNp membrane surface was relatively hydrophilic. This discrepancy was probably attributed to the fact that the nanopores were generated after nonpolar chloroform evaporation, while the surface was solidified in methanol and had more exposed polar groups. A good hydrophobic–hydrophilic balance and a good distribution of membrane surface, derived from homogeneously nonpolar phenyl and methyl groups and polar cyanides and oxygen heterocycles, might be the reasons for the weakened adhesiveness and coagulation of AsNp, like amphiphilic polymers [26,27]. Besides the hydrophobic–hydrophilic nature, the repelling of the negatively charged surface, as identified by Zeta potential data (Fig. 3H), toward blood components with the same charged property contributed to hemocompatibility as well. Additionally, the smoother surface of AsNp than that of PSF and PDMS and other porous membranes, which could minimize contact area and shear force to blood components, was another important factor for ameliorating antithrombosis and antihemolysis (Fig. 1G and Fig. S13C and D) [3,26]. Thrombosis and hemolysis are complex and difficult to fully understand. Nevertheless, the data presented here confirmed the hemocompatibility of the AsNp membrane.

### Fast blood oxygenation

**Fig. 4.**
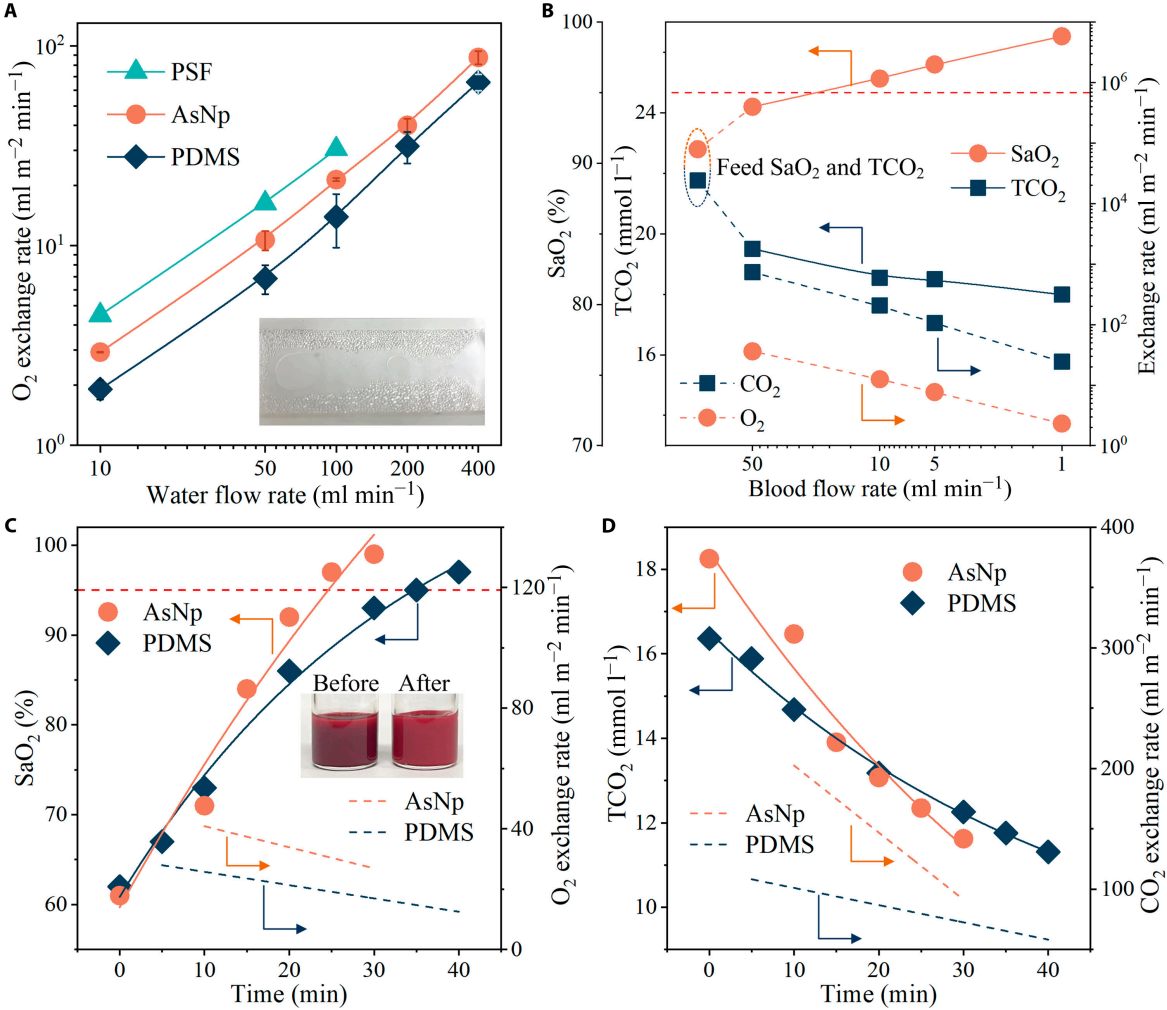
Blood oxygenation performance. (A) Water oxygenation performance of the PSF, PDMS, and AsNp membranes. Inset photograph shows the water leakage of PSF during operation. (B) Oxygen saturation (SaO_2_), total CO_2_ concentration (TCO_2_), O_2_ exchange rate, and CO_2_ exchange rate of the AsNp membrane at different blood flow rates. (C) Blood SaO_2_ and O_2_ exchange rate of the PDMS and AsNp membranes with oxygenation circulation time extending. Inset photographs present the color change of blood before and after oxygenation circulation through the AsNp membrane. (D) Blood TCO_2_ concentration and CO_2_ exchange rate of the PDMS and AsNp membranes as oxygenation time extending. Blood was circulated with a flow rate of 50 ml min^−1^.

The gas exchange of the membranes was evaluated by oxygenation using water and porcine blood (Fig. S14). With a water flow acceleration from 10 to 400 ml min^−1^, the O_2_ exchange rate of the AsNp membrane averaged from 3 samples changed from 3 to 90 ml m^−2^ min^−1^ and was greater than that of PDMS (Fig. 4A). Notably, the leakage of the PSF membrane occurred due to wetting after an operation lasting tens of minutes (Fig. 4A, inset). Originating from the better gas solubility in blood, the O_2_ exchange rate of the AsNp membrane for porcine blood with an oxygen saturation (SaO_2_) of about 90% and a flow rate of 1 to 50 ml min^−1^ was larger than that for deionized water with the same flow rates and reached 2 to 36 ml m^−2^ min^−1^ (Fig. 4B). Correspondingly, the CO_2_ in blood was removed with a transport rate of 20 to 735 ml m^−2^ min^−1^ owing to preferential solubility and permselectivity (Fig. 4B). It was clear that blood, after making contact with the membrane, displayed elevated O_2_ and decreased CO_2_ concentrations (Fig. S15). Since a slower flow rate resulted in a longer dwell time, the increment and decrement of O_2_ and CO_2_ concentrations became more obvious as flow rate decreased. At 1 ml min^−1^, the SaO_2_ for the AsNp membranes increased to 99%, while the total CO_2_ concentration (TCO_2_) dropped from 21.8 to 18.0 mmol l^−1^.

Further, the blood circulation was carried out with a SaO_2_ of 60% ± 5% to simulate oxygenation for venous blood (Fig. 4C and D and Figs. S16 and S17). As circulation time prolonged, the blood was oxygenated and the SaO_2_ increased to over 95%, which was similar to that of arterial blood. Comparatively, the AsNp membrane required a shorter oxygenation period of 25 min and exhibited a faster O_2_ exchange rate of 20 to 60 ml m^−2^ min^−1^ than PDMS with values of 35 min and 10 to 30 ml m^−2^ min^−1^. With a blood circulation of 30 and 40 min for AsNp and PDMS with an initial TCO_2_ (pCO_2_) of 18.3 mmol l^−1^ (70.1 mmHg) and 16.4 mmol l^−1^ (60.7 mmHg), the TCO_2_ (pCO_2_) substantially reduced to 11.6 mmol l^−1^ (26.8 mmHg) and 11.3 mmol l^−1^ (26.4 mmHg), accompanied by a CO_2_ removal rate of 100 to 350 and 60 to 160 ml m^−2^ min^−1^, respectively. Rapid CO_2_ transport might be useful for mitigating CO_2_ toxic effects. A downward trend of gas exchange rates over time was ascribed to the gradually weakened driving force for exchange due to the CO_2_ removal and O_2_ accumulation. Distinct from the CO_2_/O_2_ selectivity of AsNp and the permeance difference (4 to 6 times) between AsNp and PDMS, the related exchange rates differed by 5 and 2 times, respectively, which were attributed to partial pressure difference and transfer resistance from blood boundary [28,29]. In general, the HGB concentration of blood is positively correlated to exchange rate. By replacing the blood with an HGB concentration of 58 g l^−1^ with another one (65 g l^−1^), at a flow rate of 50 ml min^−1^, the O_2_ and CO_2_ exchange rates of AsNp were enhanced to 170 and 740 ml m^−2^ min^−1^, respectively (Fig. S18). It was worth emphasizing that the parameters of membrane modules and operations affected gas transports immensely. Under similar conditions, the commercial PMP membranes had an O_2_ exchange rate of 10 ml m^−2^ min^−1^ as reported in the literature [14]. In light of more HGB in the human blood and the rational parameters of the oxygenator, the AsNp membrane is expected to have a higher efficiency during practical application. Furthermore, the clean surface after blood circulation verified the hemocompatibility of the AsNp membrane (Fig. S19).

## Discussion

Nanoporous membranes have been demonstrated with great potential for molecular separation, but no effort about their utilization in artificial organs has been reported. We have fabricated an AsNp membrane by simple NIPS of PIM-1 and demonstrated its great performance for blood oxygenation application, with a restricted protein adsorption of 10.5 μg cm^−2^, a low HR of 0.29%, and fast CO_2_ and O_2_ exchange rates of 100 to 350 and 20 to 60 ml m^−2^ min^−1^, respectively. It has been found that the properties of the AsNp membrane including superhydrophobic nanopores, asymmetric structure, hydrophobicity and hydrophilicity, electronegativity, and surface smoothness are suitable for gas permeation, oxygenation exchange, leakage inhibition, antithrombosis, and antihemolysis. Overall, even though designing a membrane oxygenator involves many complex factors and systematic clinical practice for patients should be further investigated [30], the AsNp membranes offer opportunities for advancing membrane-based artificial lungs. The concepts of fabrication and application of nanoporous membranes will provide an alternative route for obtaining high-performance membranes and expand their application scope in artificial organs.

## Materials and Methods

### Chemicals and reagents

Anhydrous potassium carbonate (K_2_CO_3_), methanol, dichloromethane, toluene, acetone, chloroform, *N-*methyl-2-pyrrolidone (NMP), polysulfone (PSF), *N*,*N*-dimethylacetamide (DMAC), polydimethylsiloxane (PDMS), tetraethyl orthosilicate, dibutyltin dilaurate, cyclohexane calcium chloride (CaCl_2_), sodium chloride (NaCl), and polytetrafluoroethylene (PTFE) substrate were purchased from Kutai Chemical Reagent Co., China. K_2_CO_3_ was dried at 393 K overnight before use. 2,3,5,6-Tetrafluoroterephthalonitrile (TFTPN) and 5,5’,6,6’-tetrahydroxy-3,3,3’,3’-tetramethyl-1,1’-spirobisindane (TTSBI) were purchased from Sigma-Aldrich. Spirobisindane was purified by crystallization in methanol and dichloromethane. Bovine serum albumin (BSA) was purchased from Biofroxx Biological Co., Germany. Sheep whole blood was purchased from Bestbio Biological Co., China. Porcine whole blood was purchased from Yuanye Biological Co., China. Calcein AM was purchased from UElandy Biological Co., China. APTT, PT, TT, and FIB reagents were purchased from Shanghai Sun Biotech Co., China.

### Characterizations

Nitrogen adsorption–desorption isotherms were collected by using a physisorption analyzer (Autosorb iQ Station 1, Quantachrome Co.) at 77 K held using a liquid nitrogen bath. The multipoint BET method and the slit-pore model-based nonlocal density functional theory method were applied to calculate the specific surface area and the pore width distribution, respectively. Water vapor isotherms were collected using the physisorption analyzer at 298 K. A field-emission scanning electron microscope (SEM, Ultra-55, Zeiss Co.) with an accelerating voltage of 5 kV was used to observe the morphology of samples. An ultrathin gold layer was coated on the prepared sample by using an ion sputter coater to minimize the recharging effect. An atomic force microscope (AFM, Bioscope Catalyst Nanoscope-V, Bruker, USA) was employed to measure the surface roughness of membranes. An ultraviolet absorption experiment was carried out by using an ultraviolet-visible (UV-vis) spectrophotometer (UV-1780, SHIMADZU). An optical contact angle and interface tension meter (ST200KB, USA KINO Industry Co.) was used to investigate the water and blood contact angles of samples. The zeta potential of membranes was measured by using a streaming potential analyzer (SurPASS, Anton Paar, Austria) with the background electrolyte of 1 mmol l^−1^ KCl solution.

### Synthesis of PIM-1

TFTPN (0.01 mol), TTSBI (0.01 mol), and K_2_CO_3_ (0.03 mol) were added into a mixture of toluene (20.0 ml) and NMP (40.0 ml) in a 3-necked flask by stirring under nitrogen atmosphere. For polycondensation of PIM-1, the temperature of the flask was increased to 433 K using an oil bath and kept for 40 min. Then, massive methanol was added to precipitate the yellow PIM-1 product and stirring continued for 3 h. After synthesis, the yellow product was isolated by centrifugation and dried at 333 K. For purification, the PIM-1 product was dissolved in chloroform, re-precipitated and stirred in methanol overnight, separated by centrifugation, and washed 3 times with deionized water and acetone. Ultimately, the prepared PIM-1 powder was dried at 373 K for 2 days.

### Preparation of the AsNp membrane

For fabrication of the AsNp membrane, the PIM-1 powder was dissolved in chloroform and stirred overnight at room temperature to obtain the polymer solution with a concentration of 5.0 wt%. To remove bubbles, the solution was stood for 12 h. A clean scraper was used to evenly cast the polymer ink on a PTFE substrate. Through standing at room temperature for a certain time (0.5, 1, 2, 5, or 10 min), the solvent was volatilized to form a skin layer. Then, the PIM-1/PTFE membrane was transferred and quenched in a nonsolvent coagulant bath at room temperature. Because of the methanol-chloroform mutual solubility and the insolubility of PIM-1 in methanol, the solvent exchange between the polymer matrix and coagulant bath induced the phase separation and PIM-1 solidification to form the AsNp membrane. After NIPS, the AsNp membrane was dried at room temperature for use.

### Preparation of the PSF membrane

For fabrication of the PSF membrane, the PSF after vacuum drying overnight was dissolved in DMAC and stirred overnight at room temperature to obtain the polymer ink with a concentration of 18 wt%. To remove bubbles, the PSF solution was stood for 12 h. A clean scraper was used to cast the polymer ink on a glass plate. After standing at room temperature for 5 s, the casted PSF layer and glass plate were immersed in deionized water for NIPS. Then, the PSF membrane was peeled off from the glass plate, washed several times with deionized water, and stored in deionized water for use.

### Preparation of the PDMS membrane

For fabrication of the PDMS/PTFE membrane, the PDMS solution with a concentration of 5 wt% was prepared with cyclohexane solvent. For crosslinking, tetraethyl orthosilicate and dibutyltin dilaurate were used as a crosslinker and catalyst, respectively. PDMS, the crosslinking agent, and the catalyst with a weight ratio of 2:2:1 were mixed in cyclohexane. Then, the PTFE substrate was soaked in the solution for 5 min and the excess solution was removed. After dip-coating, the PDMS/PTFE membrane was thermally treated at 80 °C for 24 h to crosslink completely. For the PDMS/PSF membrane, the preparation procedures were similar to those for the PDMS/PTFE membrane, except that the PTFE substrate was replaced by the PSF membrane. It should be noted that the PDMS membrane in the manuscript usually represented the PDMS/PTFE membrane, unless otherwise specified.

### Gas permeation measurement

The gas permeation property of the prepared membranes was assessed at room temperature. A membrane with an effective area of 3.14 cm^2^ was sealed in a permeation cell by an O-ring. CO_2_, O_2_, or N_2_ was injected into the feed side with a transmembrane pressure of 0.1 MPa. After running steady, the permeate gas was collected and recorded to evaluate the gas transport.

Permeance (*P*, GPU, 1 GPU = 3.35 × 10^−10^ mol m^2^ s^−1^ Pa^−1^) was calculated using Eq. 1:

$$P=\frac{1}{p_{u}-p_{d}}\times \frac{1}{A}\times \frac{p_{d}\times \mathrm{\Delta V}}{R\times \left(273.15\right)\times \mathrm{\Delta t}}$$ (1)

where *p_u_* and *p_d_* are the upstream and downstream pressures, respectively. *A* is the effective membrane area. Δ*V* and Δ*t* are the permeate volume and time of gas through the membrane, respectively. *R* and *T* are the gas constant value and temperature, respectively.

Permeability (*P_er_*, Barrer, 1 Barrer = 3.35 × 10^−16^ m mol m^−2^ s^−1^ Pa^−1^) was calculated using Eq. 2:

$$P_{\mathrm{er}}=\frac{1}{p_{u}-p_{d}}\times \frac{1}{A}\times \frac{p_{d}\times \mathrm{\Delta V}}{R\times \left(273.15\right)\times \mathrm{\Delta t}}$$ (2)

where *l* is the membrane thickness.

Selectivity (*S*) was calculated using Eq. 3:

$$S=\frac{P_{i}}{P_{j}}$$ (3)

where *P_i_* and *P_j_* are permeances of gas *i* and *j*, respectively.

### Water permeation measurement

The water permeation property of the prepared membranes was measured at room temperature by using a cross-flow filtration system. A membrane with an effective area of 7.0 cm^2^ was sealed in a permeation cell by an O-ring. Water was injected into the feed side with a transmembrane pressure of 0.1, 0.2, 0.3, or 0.4 MPa and a cross-flow rate of 30.0 l h^−1^. After running steady, the permeate water was collected and recorded to calculate flux.

Water flux (*F*, L m^−2^ h^−1^) was calculated using Eq. 4:

$$F=\frac{\mathrm{\Delta V}}{A\cdot \mathrm{\Delta t}}$$ (4)

where Δ*V* and Δ*t* are the permeate volume and time of water through the membrane, respectively. *A* is the effective membrane area.

### Protein adsorption

Before measurement, the membrane sample with an area of 1 × 1 cm^2^ was immersed in phosphate buffer solution (PBS) solution (pH 7.4) for 24 h to remove possible impurities. For protein adsorption, the membrane was incubated in BSA PBS solution with a concentration of 1.0 mg ml^−1^ at 310 K for 2 h. After soaking, the membrane sample was taken out and washed with PBS solution to remove non-adsorbed protein. Subsequently, the membrane sample was immersed in sodium dodecyl sulfate (SDS) solution with a concentration of 2 wt% and subjected to ultrasonication for 1 h to dissolve the adsorbed protein on the membrane surface. More than 95% of the adsorbed protein could be eluted into SDS solution. Ultimately, the amount of BSA was measured by using a UV-vis spectrophotometer to study the adsorption.

### Platelet adhesion and activation

Platelet adhesion and activation were investigated by using a fluorescence microscope and SEM. Sheep whole blood (10 ml) was centrifuged at 1,000 rpm for 15 min and the supernatant was separated to obtain platelet-rich plasma (PRP). For platelet adhesion test, the membrane sample with an area of 1×1 cm^2^ was incubated in PRP (500 μl) and was shaken at 100 rpm with a temperature of 310 K for 2 h. After incubation, the membrane sample was taken out and slightly rinsed with PBS to remove the non-adherent platelets. For fluorescence staining, the membrane sample was incubated in Calcein-AM PBS (2 μM) solution at 310 K for 30 min and rinsed with PBS. A fluorescence microscope (490/515 nm) was used to observe the platelet aggregation on the membrane. For platelet activation, the membrane sample with an area of 1×1 cm^2^ was immersed in PBS solution at 310 K for 1 h and incubated in PRP (500 μl) at 310 K for 2 h. After incubation, the membrane sample was taken out and slightly rinsed by PBS solution to remove the non-adherent platelets. The membrane sample was immersed in glutaraldehyde solution with a concentration of 2.5 wt% at 277 K for 24 h to fix the adhered platelets. After immersion, the membrane sample was rinsed with PBS solution and dehydrated through a series of gradient concentrations of alcohol/PBS solutions (25%, 50%, 75%, 95%, and 100%) for 15 min each. After dehydration, the platelet adhesion and activation on membrane was observed using SEM.

### HR measurement

A membrane sample with an area of 1 × 1 cm^2^ was rinsed 3 times with deionized water and normal saline, and then was immersed in normal saline (10 ml) at 310 K for 30 min. Sheep whole blood (8.0 ml) was diluted by normal saline (10 ml), and the diluted whole blood (200 μl) was dropped into the above normal saline containing the soaked membrane sample. After incubation of the membrane sample at 310 K for 60 min, the normal saline containing diluted whole blood was treated by centrifugation at 2,500 rpm for 5 min. Then, the supernatant was separated and its absorbance was measured by using a UV-vis spectrophotometer at 545 nm. Meanwhile, the diluted whole blood (200 μl) was directly added into deionized water (10 ml) or normal saline (10 ml), and the absorbance of the supernatant from centrifugation was measured as a positive or negative control, respectively.

HR was calculated using Eq. 5:

$$\mathrm{HR}\left(\%\right)=\frac{\mathrm{HS}-\mathrm{HN}}{\mathrm{HP}-\mathrm{HN}}$$ (5)

HS represents the absorbance of sample. HN and HP represent the absorbance of negative control and positive control, respectively.

### BCI measurement

A membrane sample with an area of 1 × 1 cm^2^ was placed at the bottom of a beaker and kept at 310 K for 5 min. Sheep whole blood (100 μl) was dropped on the membrane surface, and the CaCl_2_ solution (10 μl, 0.2 mol·l^−1^) was added to the membrane surface as well. After incubation at 310 K for a certain time (5, 10, 15, or 20 min), the deionized water (25 ml) was added in the beaker and was shaken at 30 rpm and 310 K for 10 min. A UV-vis spectrophotometer was used to measure the absorbance of the solution at 545 nm. Meanwhile, sheep whole blood (100 μl) was directly added into the deionized water (25 ml) and its absorbance was measured using a UV-vis spectrophotometer as a control value.

BCI was calculated using Eq. 6:

$$\mathrm{BCI}\left(\%\right)=\frac{I_{s}}{I_{w}}\times 100$$ (6)

*I_s_* represents sample absorbance. *I_w_* represents control absorbance.

### Anticoagulation measurement

Activated partial thromboplastin time (APTT), prothrombin time (PT), thrombin time (TT), and fibrinogen (FIB) were measured by a semi-automatic blood coagulation analyzer (MTN-II, matenu) to evaluate the anticoagulant properties of the membrane. Sheep whole blood (10 ml) was centrifuged at 3,000 rpm for 15 min and the supernatant was separated to obtain platelet-poor plasma (PPP). The membrane sample with an area of 0.5 × 0.5 cm^2^ was incubated in PPP (100 μl) with a temperature of 310 K for 30 min. After incubation, the incubated PPP (50 μl) and APTT agent (50 μl) were added into a test cup. After incubation at 310 K for 3 min, the CaCl_2_ solution (50 μl, 0.025 mol·l^−1^, incubated at 310 K for 10 min before using) was added, and then the APTT was measured. For PT test, the incubated PPP (50 μl) was added into a test cup at 310 K for 3 min and the PT agent (100 μl, incubated at 310 K for 10 min before using) was added, and then the PT was measured. For the TT test, the incubated PPP (100 μl) was added into a test cup at 310 K for 3 min and a TT agent (100 μl) was added, and then the TT was measured. For the FIB test, the incubated PPP (50 μl) was diluted by the FIB buffer solution (450 μl) and the diluted PPP (50 μl) was added into a test cup. After incubation at 310 K for 3 min, the FIB thrombin solution (50 μl) was added, and then the FIB content of PPP was calculated based on a standard curve.

### Water oxygenation

The water oxygenation performance of the prepared membranes was evaluated by using a self-made apparatus shown in Fig. S7. A membrane with an effective area of 3.5 × 10 cm^2^ was sealed into a rectangular membrane module. Deionized water was deoxygenated by N_2_ with a flow rate of 160 ml min^−1^ to reduce O_2_ concentration below 1.0 mg l^−1^, and was injected into one side of the membrane module with a flow rate of 10, 50, 100, 200, or 400 ml min^−1^. For oxygenation, O_2_ was input into the other side of the membrane module with a flow rate of 200 ml min^−1^. Correspondingly, the flow rate ratios of feed gas to liquid were 20:1, 4:1, 2:1, 1:1, and 0.5:1. Oxygenation system temperature was maintained at 310 K. Water O_2_ concentration was measured by using a dissolved oxygen meter (JPB-607A, Leici). The oxygen exchange rate of the membranes was calculated by the O_2_ concentration of the water before and after oxygenation.

The oxygen exchange rate for water (*R_W_*, ml m^−2^ min^−1^) was calculated using Eq. 7:

$$R_{W}=\frac{Q_{W}\left(c_{a}-c_{b}\right)}{A}$$ (7)

where *Q_W_* (l min^−1^) is the flow rate of water. *c_b_* (ml l^−1^) and *c_a_* (ml l^−1^) are the O_2_ concentrations of solutions before and after oxygenation, respectively. *A* (m^2^) is the effective membrane area.

### Blood oxygenation

Similar to water oxygenation, blood oxygenation of the prepared membranes was investigated by using the self-made apparatus. Animal porcine whole blood (500 ml) was firstly deoxygenated by the N_2_ and CO_2_ mixture with a ratio of 9:1 and a flow rate of 50 ml min^−1^ to obtain the blood with an oxygen saturation (SaO_2_) of about 90%. To reduce the hemolysis from flow shear force, porcine blood was injected into one side of the membrane module at a flow rate of 50, 10, 5, or 1 ml min^−1^. Meanwhile, O_2_ was injected into the other side of the membrane module with a flow rate of 200 ml min^−1^. Correspondingly, the flow rate ratios of feed gas to liquid were 4:1, 20:1, 40:1, and 200:1. The oxygenation system temperature was maintained at 310 K and the membrane with an effective area of 3.5 × 10 cm^2^ was sealed in the membrane module. Blood sample was taken at the inlet and outlet of the membrane module. A blood gas analyzer (i-STAT G300, Abbott) was employed to analyze HGB concentration, O_2_ partial pressure, oxygen saturation, CO_2_ partial pressure, HCO_3_^−^, total CO_2_ concentration, etc. of blood before and after oxygenation.

The oxygen concentration (*c*O_2_) of blood was calculated using Eq. 8:

$$cO_{2}=c_{\mathrm{Hb}}\times 1.34o{\mathrm{SaO}}_{2}+pO_{2}\times 0.03$$ (8)

where *c*O_2_ (ml l^−1^) is the O_2_ concentration of blood. *c_Hb_* (g l^−1^) is HGB concentration. SaO_2_ is the oxygen saturation of blood. *p*O_2_ (mmHg) is the partial pressure of O_2_. 1.34 is the amount in milliliters of O_2_ that could be carried by 1.0 g of HGB. 0.03 is the amount in milliliters of O_2_ that could be dissolved for each 1 mmHg per liter of blood.

Oxygen exchange rate (*R_O2,1_*, ml m^−2^ min^−1^) was calculated using Eq. 9:

$$R_{O2,1}=\frac{Q_{b}\left(cO_{2,a}-cO_{2,b}\right)}{A}$$ (9)

where *Q_b_* (l min^−1^) is the blood flow rate. *c*O_2*,b*_ (ml l^−1^) and *c*O_2,*a*_ (ml l^−1^) are the O_2_ concentrations of blood before and after oxygenation at the inlet and outlet of the membrane module, respectively. *A* (m^2^) is the effective membrane area.

Carbon dioxide exchange rate (*R_CO2,1_*, ml m^−2^ min^−1^) was calculated using Eq. 10:

$$R_{\mathrm{CO}2,1}=\frac{Q_{b}\left({\mathrm{TCO}}_{2,b}-{\mathrm{TCO}}_{2,a}\right)\times 22.4}{A}$$ (10)

where *Q_b_* (l min^−1^) is the flow rate of blood. TCO_2,*b*_ (mmol l^−1^) and TCO_2,*a*_ (mmol l^−1^) are the total CO_2_ concentrations of blood before and after oxygenation at the inlet and outlet of the membrane module, respectively. *A* (m^2^) is the effective membrane area.

### Blood oxygenation circulation

Oxygenation circulation was carried out by using the self-made apparatus. Animal porcine whole blood (100 ml) was firstly deoxygenated by the N_2_ and CO_2_ mixture with a ratio of 9:1 and a flow rate of 50 ml min^−1^ to obtain the blood with an oxygen saturation (SaO_2_) of 60% ± 5%. Porcine blood with a flow rate of 50 or 400 ml min^−1^ was circulated from a reservoir tank to a membrane module with an effective membrane area of 3.5 × 10 cm^2^ and then placed back to the tank. Meanwhile, O_2_ was injected into the membrane module with a flow rate of 200 ml min^−1^. Correspondingly, the flow rate ratios of feed gas to liquid were 4:1 and 0.5:1. Oxygenation system temperature was held at 310 K. A blood gas analyzer (i-STAT G300, Abbott) was employed to monitor the HGB concentration, O_2_ partial pressure, oxygen saturation, CO_2_ partial pressure, HCO_3_^−^, total CO_2_ concentration, etc. of the blood in the reservoir.

Oxygen exchange rate (*R_O2,2_*, ml m^−2^ min^−1^) was calculated using Eq. 11:

$$R_{O2,2}=\frac{d\left({\mathrm{cO}}_{2}\right)\cdot V}{\mathrm{dt}\cdot A}$$ (11)

where *c*O_2_ (ml l^−1^) is the O_2_ concentration of the blood in the reservoir. *V* (l) is the blood volume. *t* (min) is the oxygenation time. *A* (m^2^) is the effective membrane area.

Carbon dioxide exchange rate (*R_CO2,2_*, ml m^−2^ min^−1^) was calculated using Eq. 12:

$$R_{\mathrm{CO}2,2}=\frac{d\left({\mathrm{TCO}}_{2}\right)\cdot 22.4\cdot V}{\mathrm{dt}\cdot A}$$ (12)

where TCO_2_ (mmol l^−1^) is the total CO_2_ concentration of the blood. *V* (l) is the blood volume. *t* (min) is the oxygenation time. *A* (m^2^) is the effective membrane area.

## Data Availability

All data are available in the main text or the supplementary materials. Additional data related to this paper may be requested from the authors.

## Supplementary Materials

Supplementary 1 Fig. S1. Nitrogen adsorption–desorption isotherms of PIM-1. Fig. S2. SEM images of the AsNp membrane with a standing time of 0.5 min. Fig. S3. SEM images of the AsNp membrane with a standing time of 2 min. Fig. S4. SEM images of the AsNp membrane with a standing time of 5 min. Fig. S5. SEM images of the AsNp membrane with a standing time of 10 min. Fig. S6. SEM images of the PTFE membrane. Fig. S7. SEM images of the PDMS (PDMS/PTFE) membrane. Fig. S8. SEM images of the PSF membrane. Fig. S9. SEM images, permeances, and selectivities of the PDMS/PSF membrane. Fig. S10. Aging behavior of the AsNp membrane after 100 and 270 days. Fig. S11. Water flux of the PSF, PDMS, and AsNp membranes. Fig. S12. Protein adsorption, hemolysis ratio (HR), blood clotting index (BCI), and fluorescent and SEM images. Fig. S13. Dynamic contact angles and AFM images. Fig. S14. Schematic of oxygenation apparatus. Fig. S15. Blood gas analysis for oxygenation with different flow rates. Fig. S16. Blood gas analysis for blood circulation with a flow rate of 50 ml min^−1^_._ Fig. S17. Oxygenation performance and blood gas analysis for blood circulation with a flow rate of 400 ml min^−1^_._ Fig. S18. Oxygenation performance and blood gas analysis of the AsNp membrane with different flow rates and an HGB concentration of 65 g l^−1^. Fig. S19. Membrane images after blood circulation.
