# Supplementary material for: Fast Blood Oxygenation through Hemocompatible Asymmetric Polymer of Intrinsic Microporosity Membranes
Source: Research (Wash D C). 2023 May 19;6:0151. doi: 10.34133/research.0151 (PMC10195972; doi:10.34133/research.0151)
Supplement: Supplementary 1 — Fig. S1. Nitrogen adsorption–desorption isotherms of PIM-1. Fig. S2. SEM images of the AsNp membrane with a standing time of 0.5 min. Fig. S3. SEM images of the AsNp membrane with a standing time of 2 min. Fig. S4. SEM images of the AsNp membrane with a standing time of 5 min. Fig. S5. SEM images of the AsNp membrane with a standing time of 10 min. Fig. S6. SEM images of the PTFE membrane. Fig. S7. SEM images of the PDMS (PDMS/PTFE) membrane. Fig. S8. SEM images of the PSF membrane. Fig. S9. SEM images, permeances, and selectivities of the PDMS/PSF membrane. Fig. S10. Aging behavior of the AsNp membrane after 100 and 270 days. Fig. S11. Water flux of the PSF, PDMS, and AsNp membranes. Fig. S12. Protein adsorption, hemolysis ratio (HR), blood clotting index (BCI), and fluorescent and SEM images. Fig. S13. Dynamic contact angles and AFM images. Fig. S14. Schematic of oxygenation apparatus. Fig. S15. Blood gas analysis for oxygenation with different flow rates. Fig. S16. Blood gas analysis for blood circulation with a flow rate of 50 ml min−1. Fig. S17. Oxygenation performance and blood gas analysis for blood circulation with a flow rate of 400 ml min−1. Fig. S18. Oxygenation performance and blood gas analysis of the AsNp membrane with different flow rates and an HGB concentration of 65 g l−1. Fig. S19. Membrane images after blood circulation. [file research.0151.f1.pdf]

Research
A SCIENCE PARTNER JOURNAL
SPJ
SCIENCE PARTNER JOURNALS

supplementary_materials

Fast Blood Oxygenation through Hemocompatible Asymmetric Polymer of Intrinsic Microporosity Membranes

Xinxi Huang†, Junping Huang†, Pengcheng Su, and Wanbin Li*

***** Address correspondence to: gandeylin@126.com

Guangdong Key Laboratory of Environmental Pollution and Health, School of Environment, Jinan University, Guangzhou, 511443, China

†These authors contributed equally to this work

This PDF file includes:

Figures S1 to S19

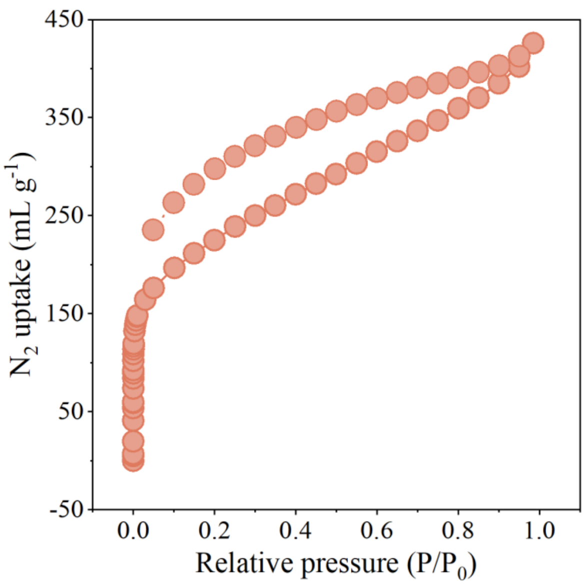

Fig. S1. Nitrogen adsorption-desorption isotherms of PIM-1. The unclosed sorption curves of MMMs were attributed to the swelling and trapping of polymers in cryogenic nitrogen.

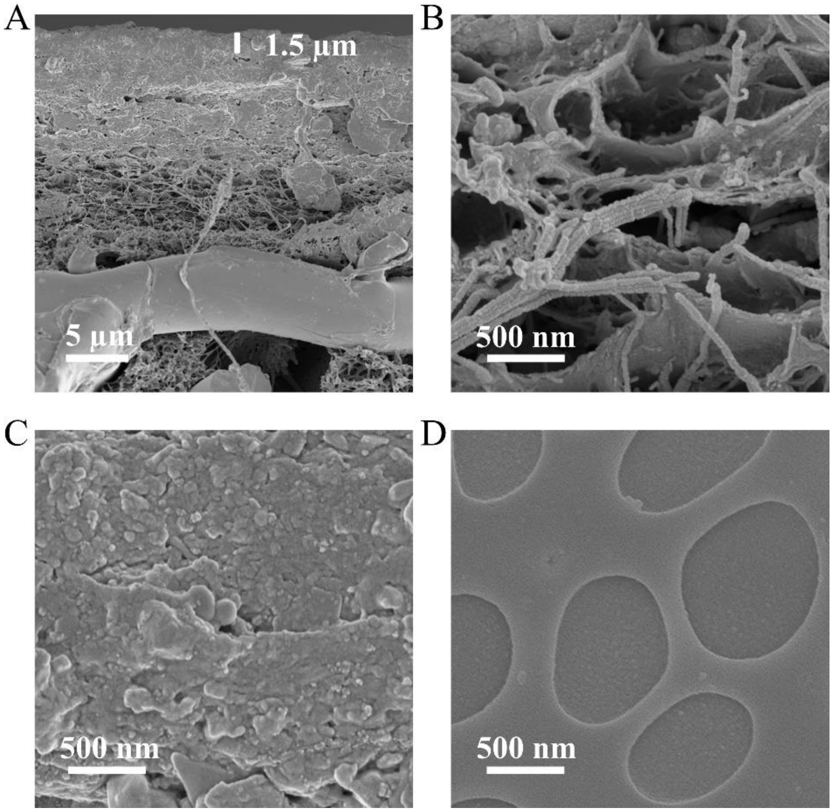

Fig. S2. SEM images of the AsNp membrane with standing time of 0.5 min. (A) Cross-sectional SEM image with low magnification. (B) Cross-sectional SEM image of porous sublayer with high magnification. (C) Cross-sectional SEM image of skin layer with high magnification. (D) Top view SEM image of the AsNp membrane. An uneven surface was attributed to the insufficient solvent evaporation and the phase separation in nonsolvent.

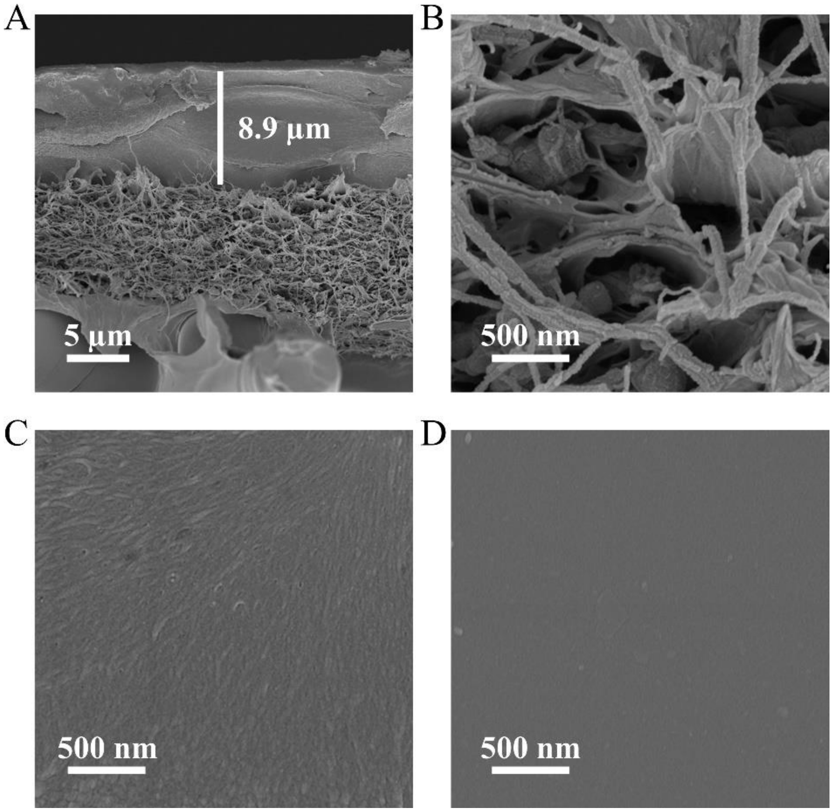

Fig. S3. SEM images of the AsNp membrane with standing time of 2 min. (A) Cross-sectional SEM image with low magnification. (B) Cross-sectional SEM image of porous sublayer with high magnification. (C) Cross-sectional SEM image of skin layer with high magnification. (D) Top view SEM image of the AsNp membrane.

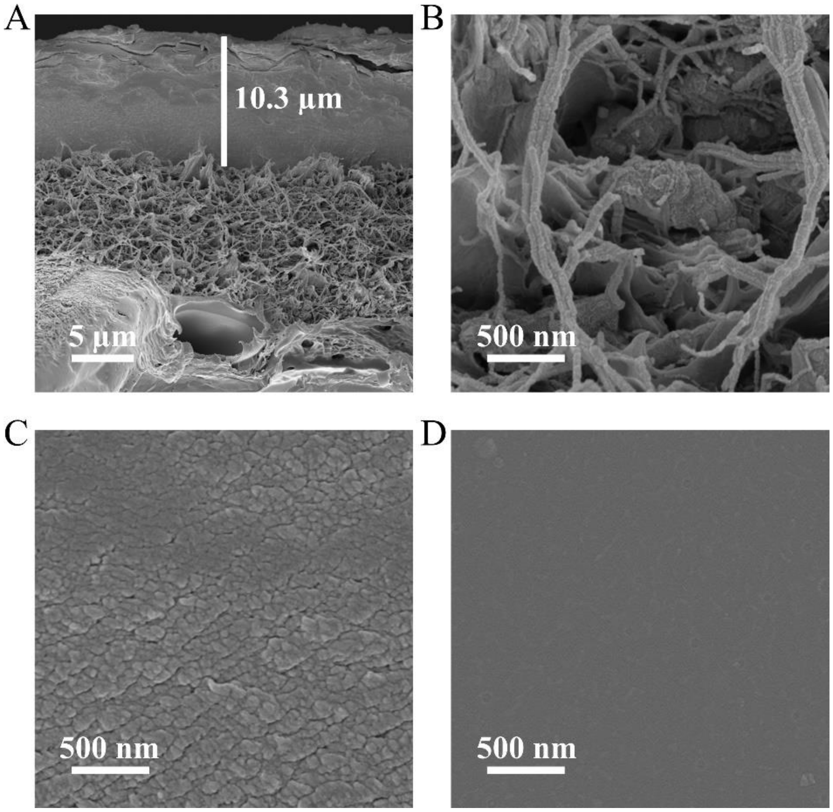

Fig. S4. SEM images of the AsNp membrane with standing time of 5 min. (A) Cross-sectional SEM image with low magnification. (B) Cross-sectional SEM image of porous sublayer with high magnification. (C) Cross-sectional SEM image of skin layer with high magnification. (D) Top view SEM image of the AsNp membrane.

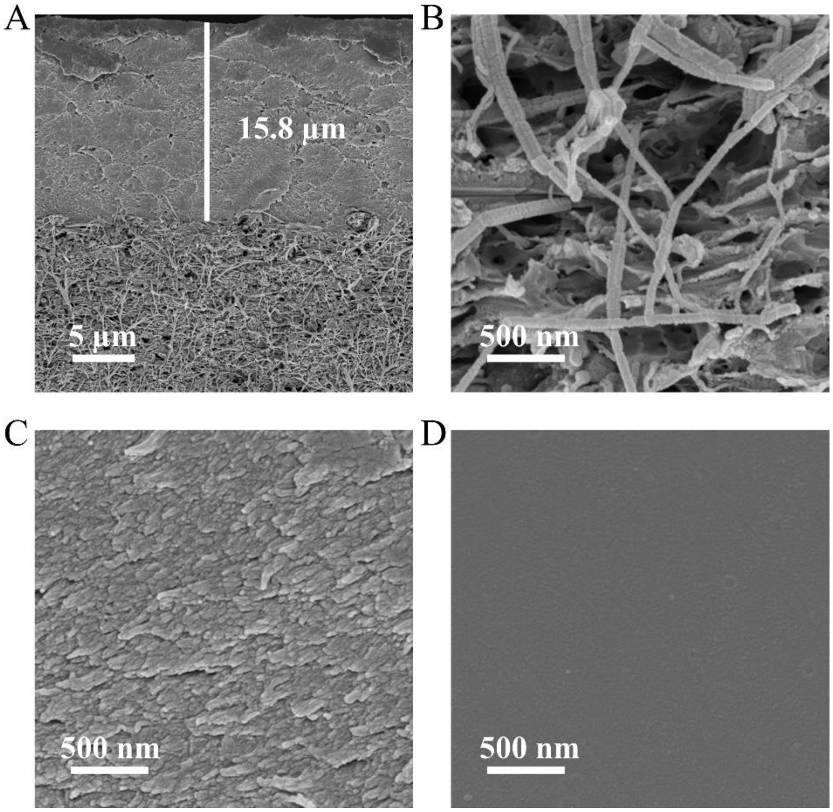

Fig. S5. SEM images of the AsNp membrane with standing time of 10 min. (A) Cross-sectional SEM image with low magnification. (B) Cross-sectional SEM image of porous sublayer with high magnification. (C) Cross-sectional SEM image of skin layer with high magnification. (D) Top view SEM image of the AsNp membrane.

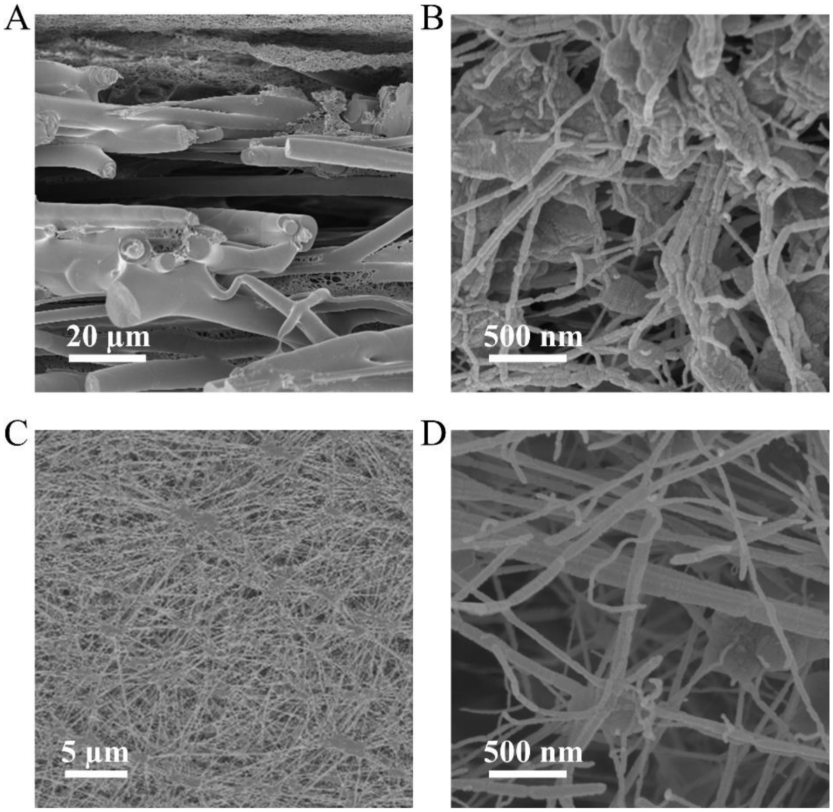

Fig. S6. SEM images of the PTFE membrane. (A and B) Cross-sectional SEM images of the PTFE substrate. (C and D) Top view SEM images of the PTFE substrate.

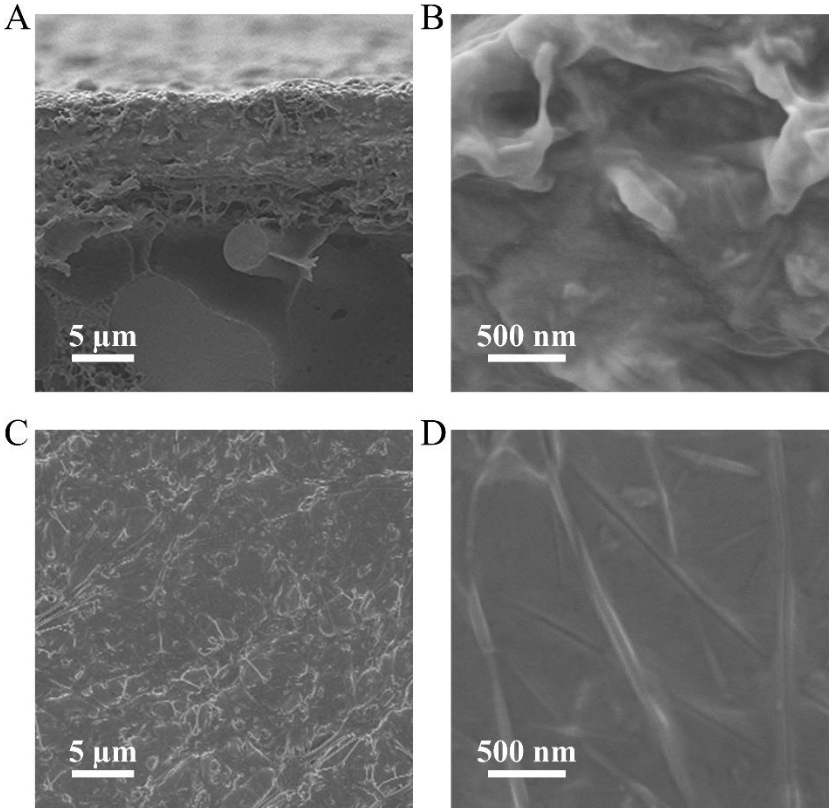

Fig. S7. SEM images of the PDMS (PDMS/PTFE) membrane. (A and B) Cross-sectional SEM images of the PDMS (PDMS/PTFE) membrane. (C and D) Top view SEM images of the PDMS (PDMS/PTFE) membrane.

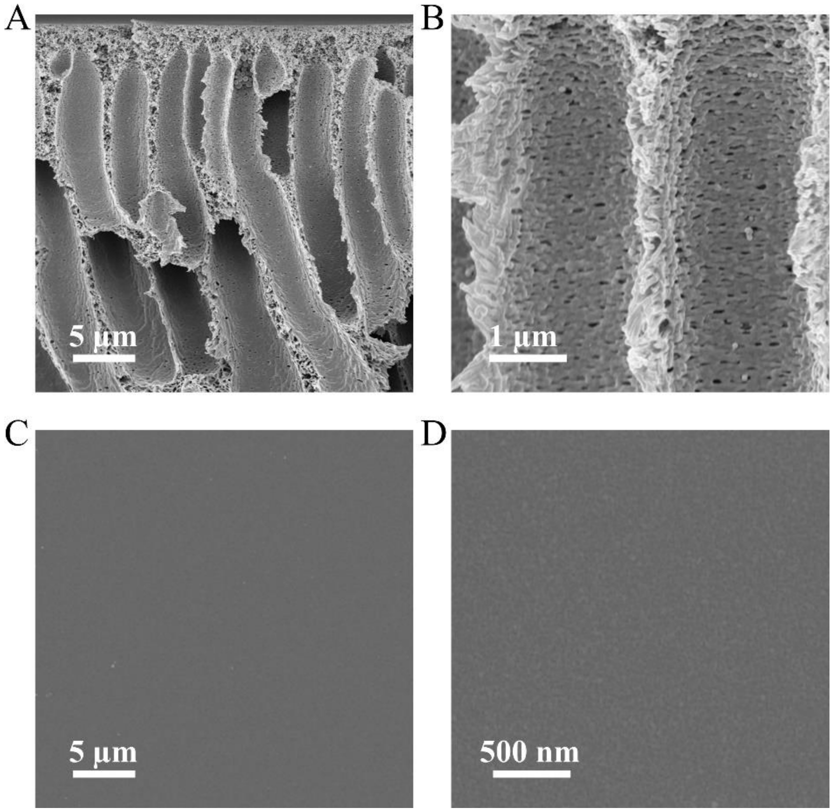

Fig. S8. SEM images of the PSF membrane. (A and B) Cross-sectional SEM images of the PSF membrane. (C and D) Top view SEM images of the PSF membrane.

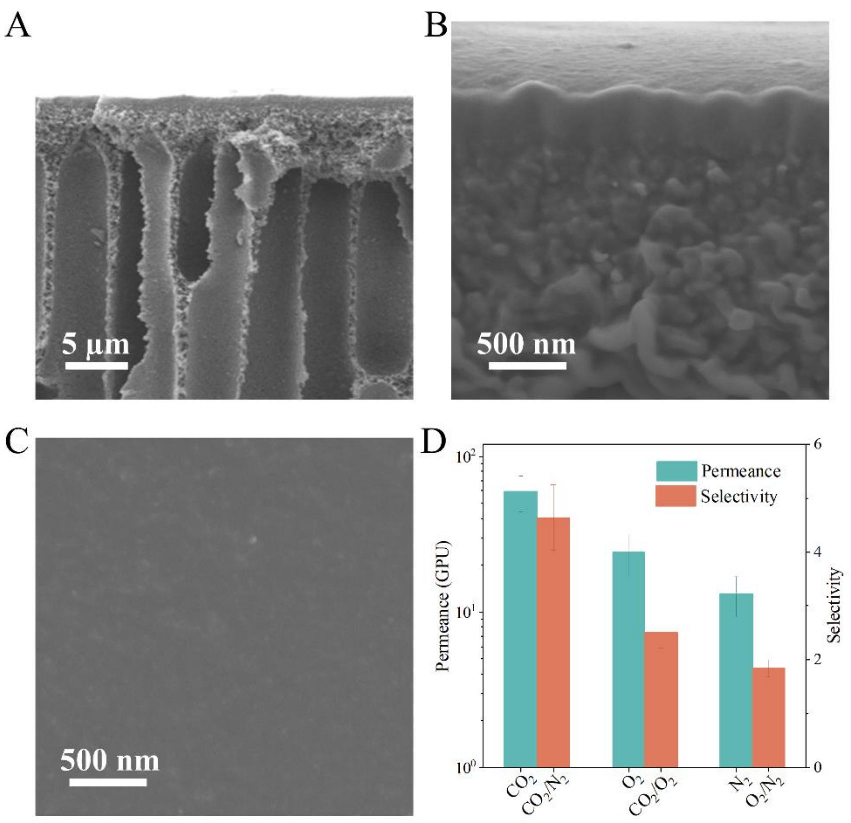

Fig. S9. SEM images, permeances, and selectivities of the PDMS/PSF membrane. (A and B) Cross-sectional SEM images of the PDMS/PSF membrane. (C) Top view SEM image of the PDMS/PSF membrane. (D) CO_2, O_2, and N_2 permeances and CO_2/N_2, CO_2/O_2, and O_2/N_2 selectivities of the PDMS/PSF membrane.

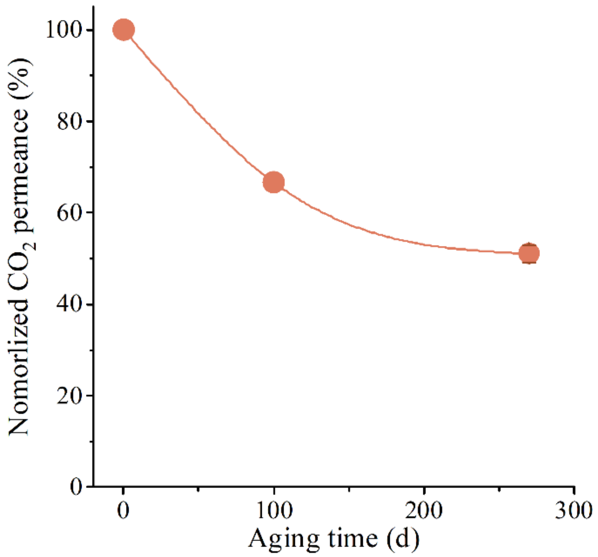

Fig. S10. Aging behavior of the AsNp membrane after 100 and 270 days. After aging for 100 and 270 days, the AsNp membrane showed CO_2 permeance reduction of 33.3% and 48.9%, respectively.

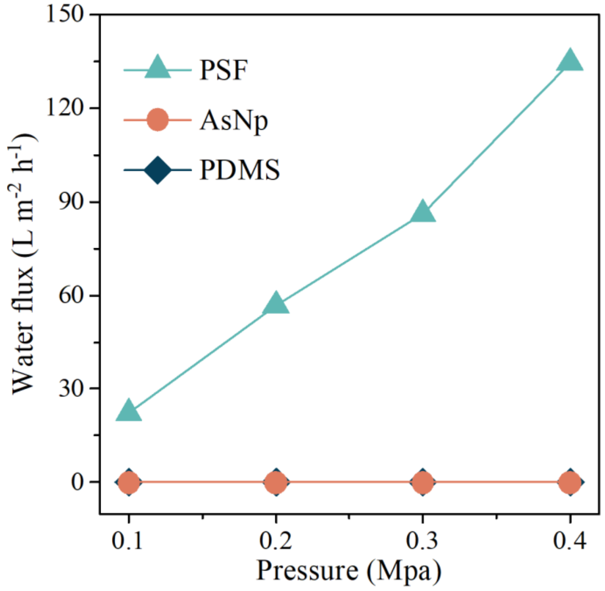

Fig. S11. Water flux of the PSF, PDMS and AsNp membranes. The PDMS and AsNp membranes were impermeable for water at different operation pressures.

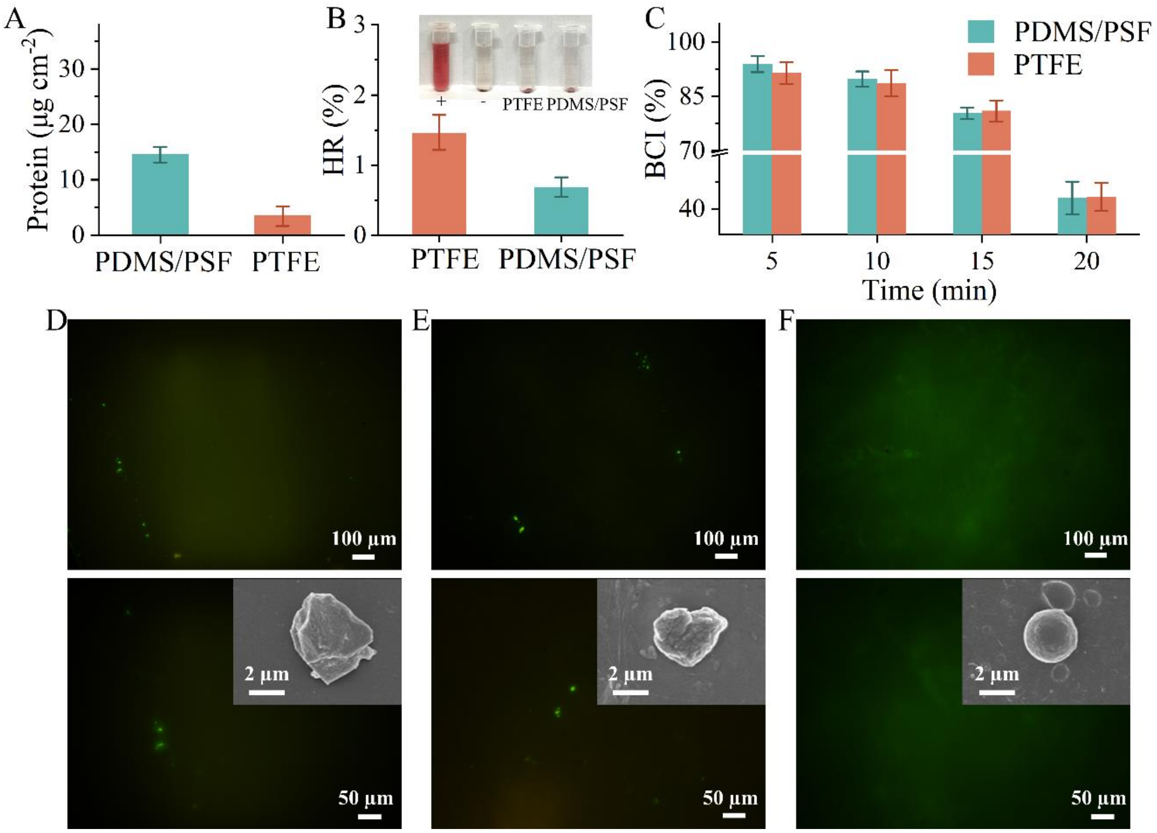

Fig. S12. Protein adsorption, hemolysis ratio (HR), blood clotting index (BCI), fluorescent and SEM images. (A) Protein adsorption of the PDMS/PSF and PTFE membranes. (B) HR of the PDMS/PSF and PTFE membranes. Insets were photographs of the positive control, negative control, and the erythrocyte solution after incubation for 1 h. (C) BCI of the PDMS/PSF and PTFE membranes at different times. (D) Fluorescent images of the PSF membrane. Inset SEM image showed the irregular platelet adhered on the PSF membrane. (E) Fluorescent images of the PDMS membrane. Inset SEM image showed the irregular platelet adhered on the PDMS membrane. (F) Fluorescent images of the AsNp membrane. Inset SEM image showed the original round platelet adhered on the AsNp membrane. Few fluorescent speckles from platelet aggregation were observed on the PSF, PDMS membranes. Relatively uniform fluorescent distribution with striped color difference was attributed to the background of the yellow AsNp membrane with the fibre-based PTFE substrate.

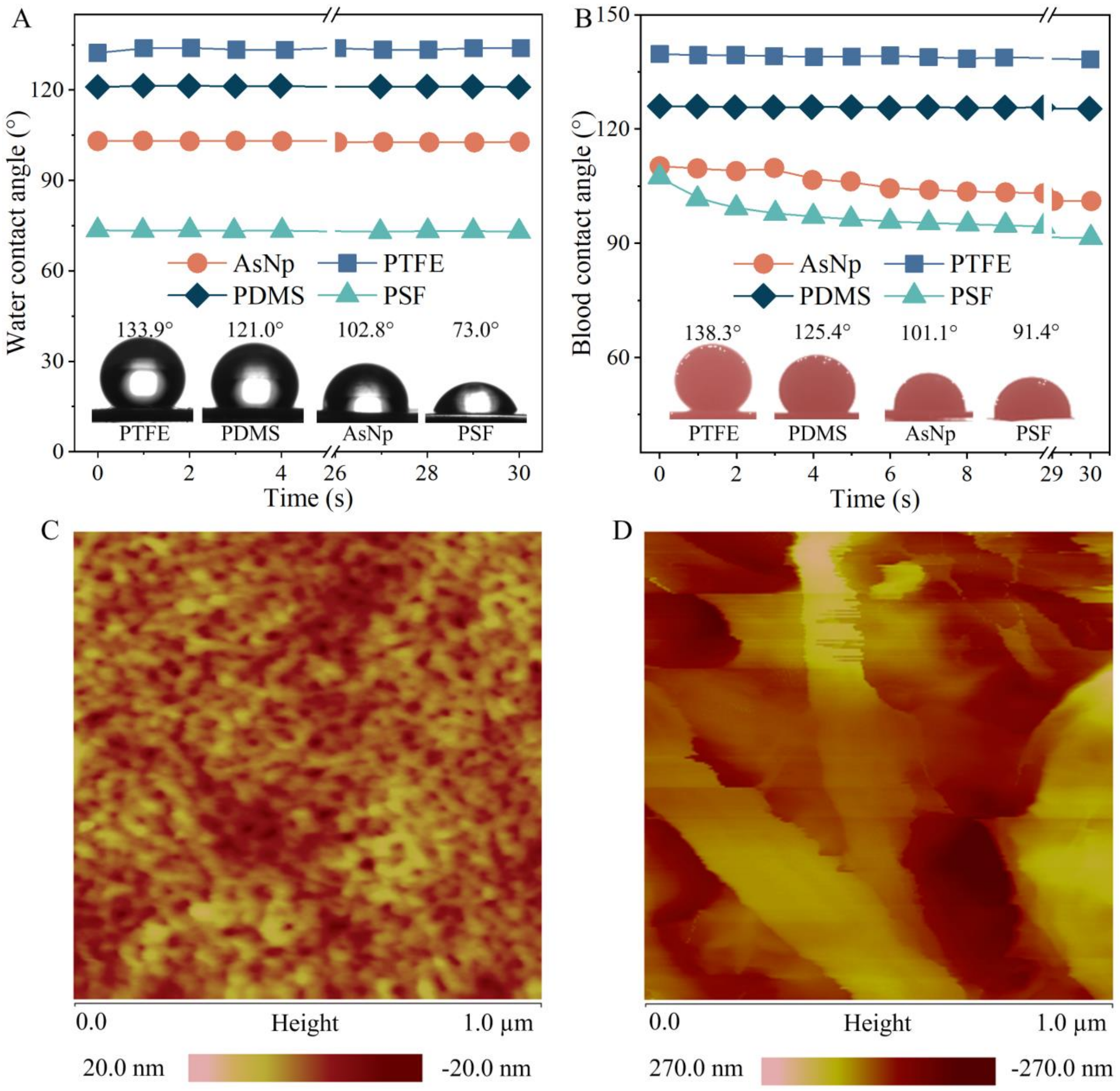

Fig. S13. Dynamic contact angles and AFM images. (A) Dynamic water contact angles of the PTFE, PDMS, PSF, and AsNp membranes. (B) Dynamic blood contact angles of the PTFE, PDMS, PSF, and AsNp membranes. (C) AFM image of the PSF membrane. (D) AFM image of the PDMS membrane. Root mean square roughness and arithmetic average roughness of the PSF membrane were 3.4 and 2.7 nm, respectively. Root mean square roughness and arithmetic average roughness of the PDMS membrane were 64 and 49 nm, respectively.

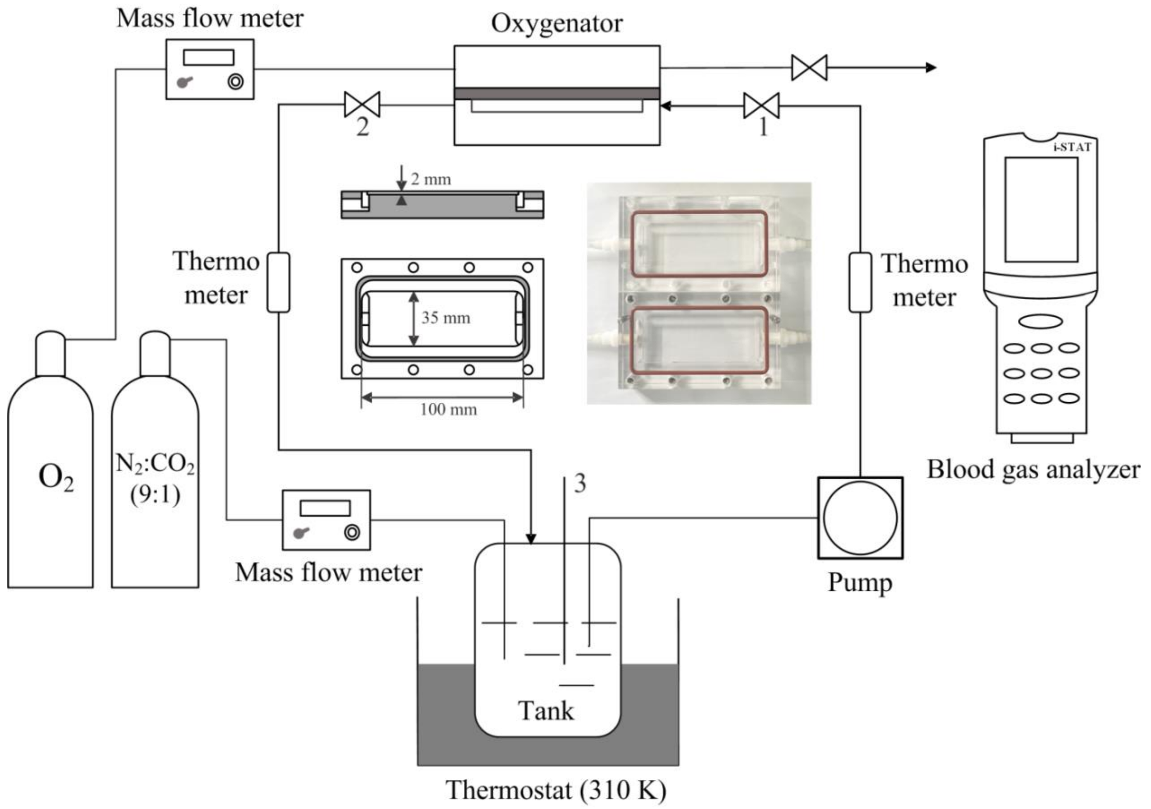

Fig. S14. Schematic of oxygenation apparatus. For water oxygenation and blood oxygenation with different flow rates, the sample was taken at the inlet (1) and outlet (2) of the membrane module. For blood circulation, the sample was taken at the blood reservoir (3). Membrane module has side volume of $0.2\times3.5\times10\ cm^3$ and effective area of $3.5\times10\ cm^2$.

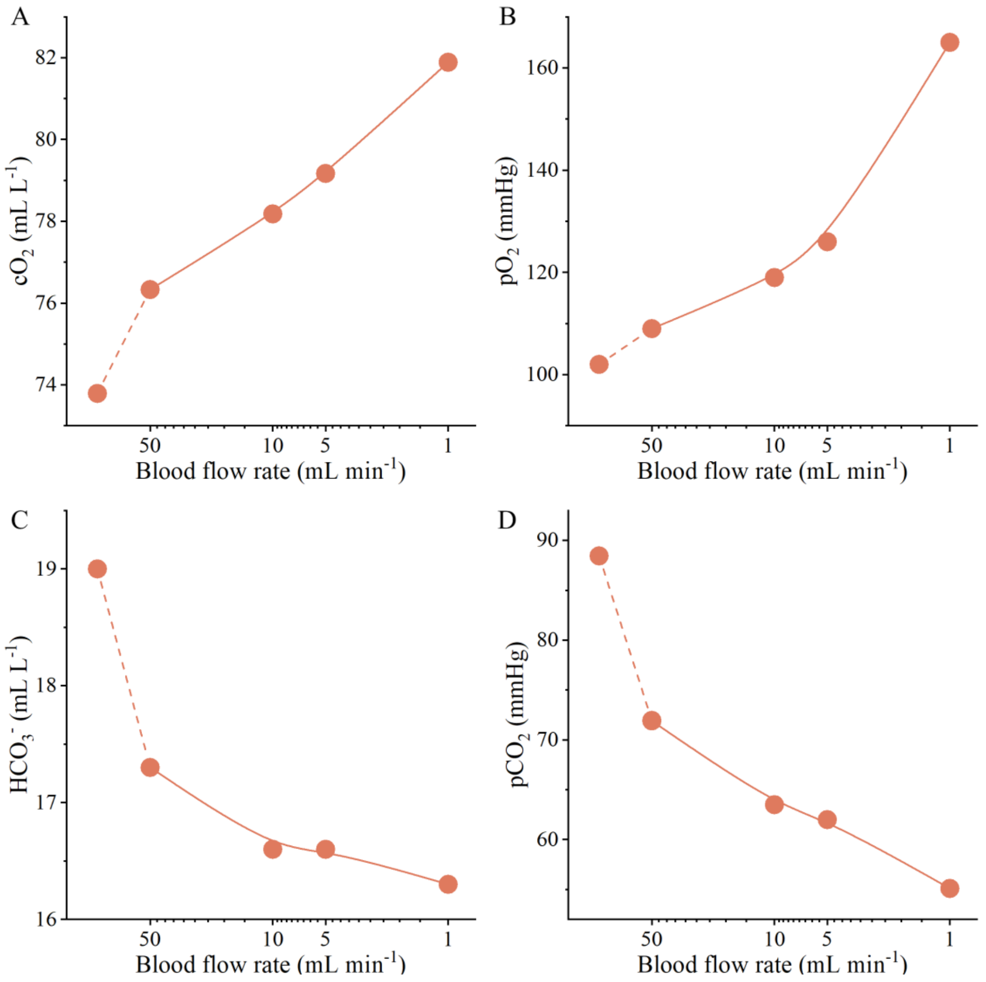

Fig. S15. Blood gas analysis for oxygenation with different flow rates. (A) O_2 concentration (cO_2) of blood before and after oxygenation with the AsNp membrane at different flow rates. (B) O_2 partial pressure (pO_2) of blood before and after oxygenation with the AsNp membrane at different flow rates. (C) HCO_3^- of blood before and oxygenation with the AsNp membrane at different flow rates. (D) CO_2 partial pressure (pCO_2) of blood before and after oxygenation with the AsNp membrane at different flow rates.

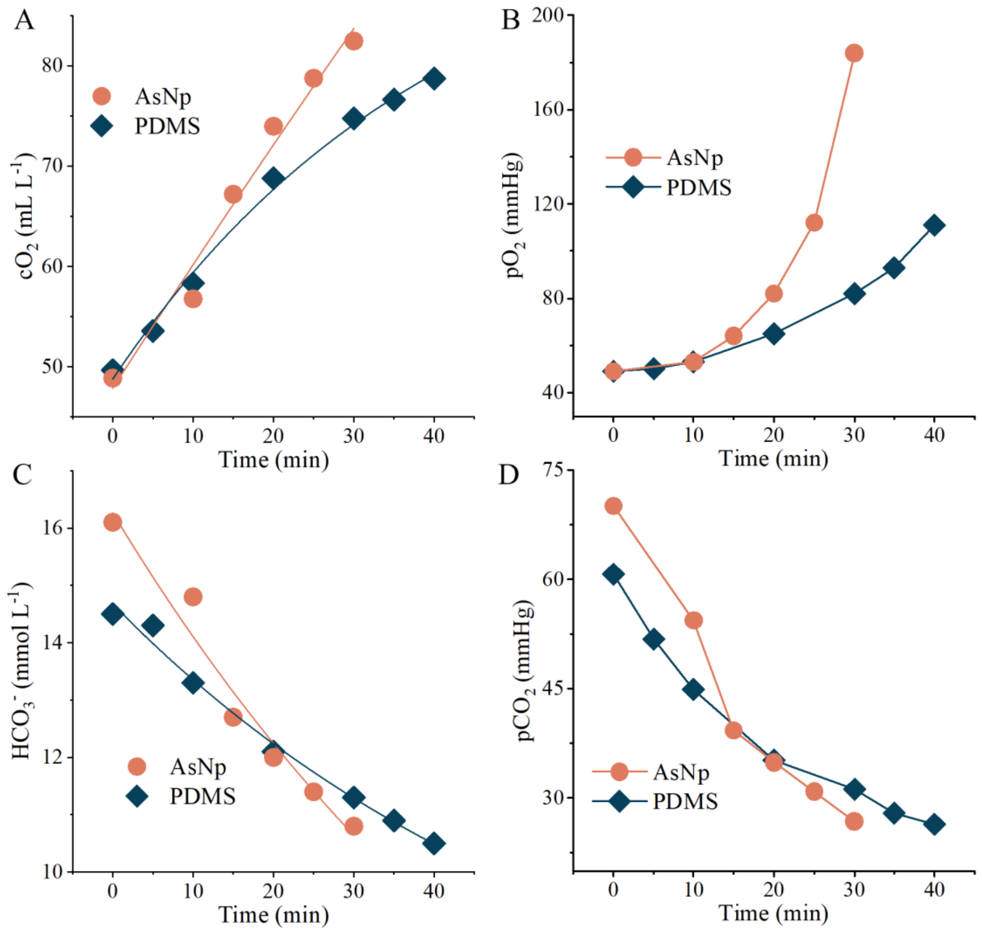

Fig. S16. Blood gas analysis for blood circulation with flow rate of 50 mL min^{-1}. (A) O_2 concentration (cO_2) of blood after oxygenation with the PDMS and AsNp membranes for different times. (B) O_2 partial pressure (pO_2) of blood after oxygenation with the PDMS and AsNp membranes for different times. (C) HCO_3^- of blood after oxygenation with the PDMS and AsNp membranes for different times. (D) CO_2 partial pressure (pCO_2) of blood after oxygenation with the PDMS and AsNp membranes for different times.

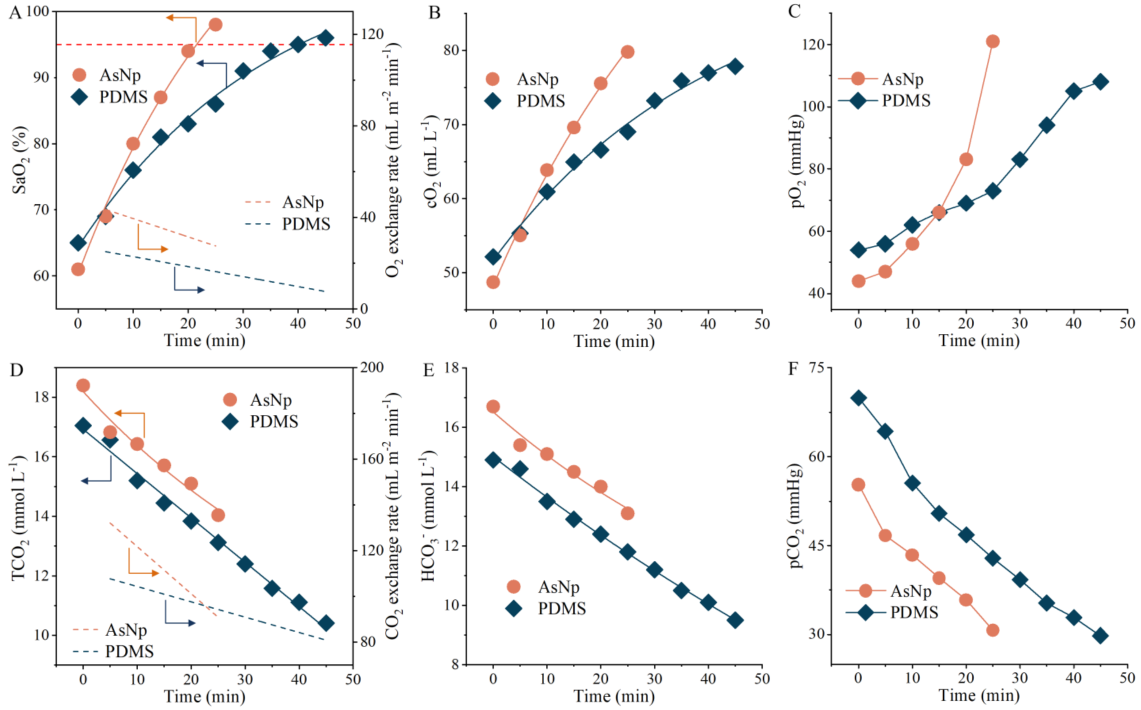

Fig. S17. Oxygenation performance and blood gas analysis for blood circulation with flow rate of 400 mL min^{-1}. (A) Oxygen saturation (SaO_2) and O_2 exchange rate of the PDMS and AsNp membranes with time extending. (B) O_2 concentration (cO_2) of blood after oxygenation with the PDMS and AsNp membranes for different times. (C) O_2 partial pressure (pO_2) of blood after oxygenation with the PDMS and AsNp membranes for different times. (D) Total CO_2 concentration (TCO_2) and CO_2 exchange rate of the PDMS and AsNp membranes with time extending. (E) HCO_3^- of blood after oxygenation with the PDMS and AsNp membranes for different times. (F) CO_2 partial pressure (pCO_2) of blood after oxygenation with the PDMS and AsNp membranes for different times.

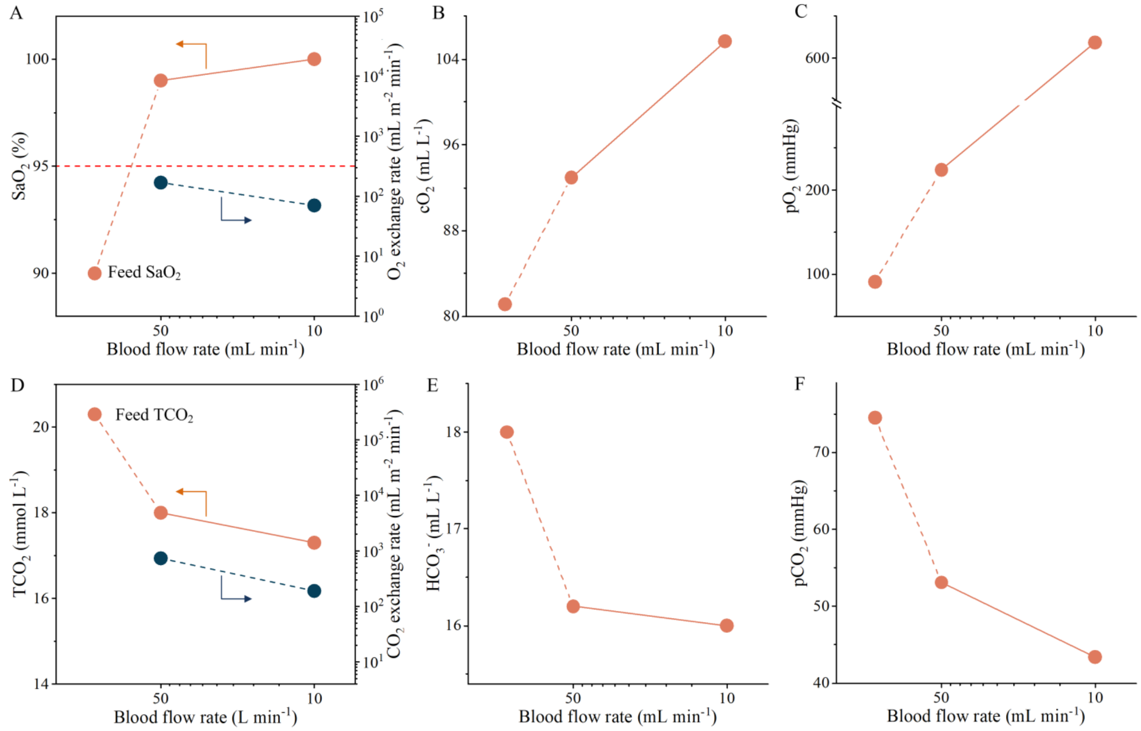

Fig. S18. Oxygenation performance and blood gas analysis of the AsNp membrane with different flow rates and HGB concentration of 65 g L^{-1}. (A) Oxygen saturation (SaO_2) and O_2 exchange rate of the AsNp membrane at different flow rates. (B) O_2 concentration (cO_2) of blood before and after oxygenation with the AsNp membrane at different flow rates. (C) O_2 partial pressure (pO_2) of blood before and after oxygenation with the AsNp membrane at different flow rates. (D) Total CO_2 concentration (TCO_2) and CO_2 exchange rate of the AsNp membrane at different flow rates. (E) HCO_3^- of blood before and oxygenation with the AsNp membrane at different flow rates. (F) CO_2 partial pressure (pCO_2) of blood before and after oxygenation with the AsNp membrane at different flow rates.

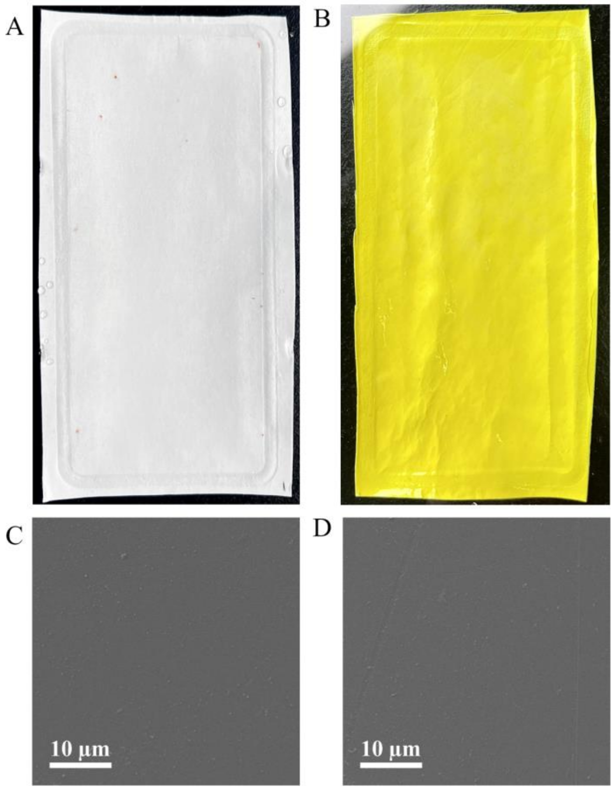

Fig. S19. Membrane images after blood circulation. (A) Photograph of the PDMS membrane after blood oxygenation circulation. (B) Photograph of the AsNp membrane after blood circulation. Few clotting spots could be observed on the PDMS membrane surface. (C and D) SEM images of the AsNp membrane after blood circulation.
